# Long Noncoding RNA CTC Inhibits Proliferation and Invasion by Targeting miR-146 to Regulate KIT in Papillary Thyroid Carcinoma

**DOI:** 10.1038/s41598-020-61577-z

**Published:** 2020-03-12

**Authors:** Baochun Liao, Shi Liu, Jiafeng Liu, Pulusu Ajay Kumar Reddy, Yong Ying, Yang Xie, Jianhua Wang, Xiangtai Zeng

**Affiliations:** 1 Department of Thyroid Surgery, First Affiliated Hospital of Gannan Medical University, Ganzhou, 341000 China; 2 State Key Laboratory of Virology, Modern Virology Research Center, College of Life Scicences, Wuhan University, Wuhan, 430072 China; 3 Department of General Surgery, Affiliated Hospital of Integrated Chinese and Western Medicine, Nanjing University of Chinese Medicine, Nanjing, 210028 China

**Keywords:** Cancer, Oncology

## Abstract

Several lines of evidence have shown that long non-coding RNAs (lncRNAs) are dysregulated in many diseases. Nevertheless, the biological relevance of the lncRNAs in papillary thyroid carcinoma (PTC) has not been fully explored. We demonstrated that CTC was a negative regulator of PTC cell migration and invasion *in vitro* and *in vivo*. We found that microRNA-146 (miR-146) is an inhibitory target of CTC. We then demonstrated that CTC functioned as a miR-146 decoy to de-repress expression of KIT. Further study demonstrated that CTC modulated the progression and chemoresistance of PTC cells via miR-146 and KIT. The analysis of hundreds of clinical specimens revealed that CTC and KIT levels were downregulated, whereas miR-146 levels were greater in PTC tissues than in normal thyroid. Their expression levels correlated with one another. In conclusion, CTC functions as a competing endogenous RNA to inhibit the progression and chemoresistance of PTC cells, and identifies CTC serve as a potential therapeutic agent to suppress PTC progression.

## Introduction

Thyroid carcinomas represent the majority of endocrine neoplasms worldwide, and their incidence has increased steadily over the past decade^1–3^. Thyroid cancers can be classified as three types: well-differentiated, poorly differentiated and anaplastic carcinomas^4,5^. Papillary thyroid carcinoma (PTC) is a well-differentiated type, accounting for 70–80% of thyroid carcinomas^4,5^. Through surgical removal, thyroid hormone and adjuvant radioactive iodine therapy, PTC has a favorable prognosis^6^. Nevertheless, some patients exhibit aggressive behavior due to metastases^6^. Therefore, identification of potential biomarkers and therapeutic targets could aid the therapy of PTC.

The non-coding RNA (ncRNA) refers to RNA molecules that cannot encode proteins, but nevertheless have biological functions^7,8^. These ncRNAs has many types, including microRNAs, snoRNAs and longer transcripts^9,10^. Long non-coding RNAs (LncRNAs) (>200 nt) are a newly-described class of RNA transcripts which had been regard as “transcriptional noise” in the past^11,12^. Nevertheless, several lines of data support the notion that particular lncRNAs function as crucial regulators in the cancer biology, rather than “nonsense fragments”^13–15^. For PTC, many functional lncRNAs have been characterized, including ATB, CASC2, H19, and NEF, suggesting that lncRNAs may be able to be diagnostic tags and potential therapeutic targets^16–20^. MircroRNAs (miRNAs) are noncoding RNA molecules in 18–25 nucleotides length that negatively regulate gene expression at the post-transcriptional level^21,22^. miRNAs regulate proliferation, metastasis and apoptosis by inhibiting tumor suppressor gene pathways or activating oncogenic pathways^23^. Almost half of human miRNAs are in the position of fragile sites and genomic regions that are associated with cancers^24^. Nevertheless, little is known about the biological role of miRNAs in PTC. A recent study reported that miR-146, miR-221 and miR-222 were upregulated in PTC compared to levels in adjacent normal tissue^25^. The target of these miRNAs appears to be c-KIT, a tyrosine kinase receptor (KIT), that is required in cell growth and differentiation^25^. These data are further corroborated by many other studies, suggesting that miR-146, miR-221 and miR-222 are molecular biomarkers for thyroid carcinoma^26–28^.

In this study, we identified a novel lncRNA, lncRNA-CTC (CTC), that regulates the proliferation and invasion of PTC cells. We also demonstrated that CTC directly binds with miR-46 and functions as an miRNA decoy to regulate KIT expression.

## Results

### LncRNA CTC inhibits the proliferation and invasion of papillary thyroid cancer cells

Because miR-146 was consider as a potential molecular biomarker in PTC^25^, we suspect that some lncRNAs regulate PTC cell via miR-146. To verify our conjecture, DIANA TOOLS (a human lncRNA target prediction tool) was used to screen lncRNAs, which have potential ability to associate with miR-146. According to the results from DIANA TOOLS, ten high-scoring candidate lncRNAs were chosen for subsequent study. Next, we compared expression levels of these potential lncRNAs in PTC tissues and the corresponding adjacent normal tissues (ANT). As shown in Supporting Fig. 1, lncRNA-CTC (CTC) was expressed at lower levels in PTC tissues than in ANT, and CTC was chosen for further study. CTC is located at chromosome 19: 27,793,496–27,799,403 with the transcript length 5908 nt (ENST00000592404). ORF (Open Reading Frame) Finder software shown that the potential ORFs for CTC were totally shorter than 480 bp (Supporting Fig. 2A). CPC (Coding Potential Calculator) software was used to identify the coding potential of CTC, and results showed that each transcript of CTC was unable to code (Supporting Fig. 2B). *In vitro* translation experiments were performed to verify these software prediction results. As shown Supporting Fig. 2C, protein bands for CTC were not observed. The 180 kD protein band of DNMT1 was used for positive control. A full-length CTC was identified using RACE experiments (Supporting Fig. 2D). The localization of CTC was also evaluated. We found that CTC was localized in both the cytoplasm and the nucleus (Supporting Fig. 2E).

**Figure 1:**
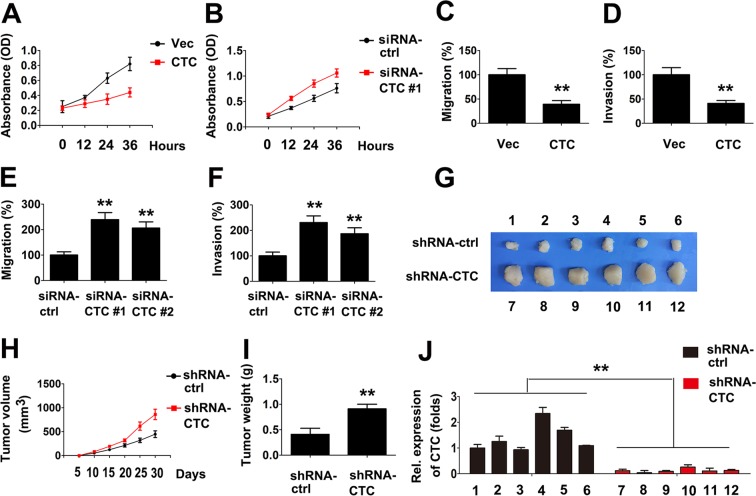
LncRNA CTC inhibits PTC cells growth and motility. (**A**) TPC-1 cells were transfected with vector control or lncRNA CTC overexpression plasmid for the indicated times prior to the cell proliferation assay. (**B**) Experiments were performed similar to those in (**D**), except the indicated siRNAs were used. (**C**,**D**) TPC-1 cells were transfected with vector control or lncRNA CTC overexpression plasmid for 36 h prior to Transwell migration (**C**) or invasion (**D**) assays. (**E**,**F**) Experiments were performed similar to those in (**C**,**D**), except indicated siRNAs were used. (**G**) Nude mice were sacrificed and photographed 1 month after subcutaneous injection. (**H**) The growth curves of tumors derived from TPC-1 cells transfected with si-CTC or si-Ctrl. (**I**) The average weight of tumors. (**J**) The relative mRNA and protein levels of lncRNA CTC in the tumor tissues from mice were detected by qRT-PCR. In the qRT-PCR experiments, the vector control was designated as 1. Bar graphs present means ± SD, n = 3 (**P < 0.01; *P < 0.05).

We next examined the potential function of CTC in the proliferation and invasion of PTC cells. To do this, we constructed overexpression plasmids and two specific small interfering RNAs (siRNAs). qRT-PCR analysis indicated that siRNA #1 and #2 inhibit the expression of CTC (Supporting Fig. 3). PTC cells proliferation rates were decreased by CTC overexpression plasmids (Fig. 1A). Conversely, knockdown of CTC increased the proliferation rates of PTC cells (Fig. 1B). In line with these data, CTC overexpression promoted PTC cells migration and invasion, whereas knockdown of CTC resulted in the inhibition of PTC cell migration and invasion (Fig. 1C–F). To determine whether CTC affect tumorigenicity *in vivo*, an additional experiment was performed. Nude mice were subcutaneously injected with CTC knockdown or control cells. We found that, compared with control mice, CTC knockdown mice exhibited higher tumor growth; however, there were no effects on body weight (Fig. 1G–I). Further experiments confirmed that CTC expression levels were higher in control mice than in CTC knockdown mice (Fig. 1J). These *in vivo* and *in vitro* data suggest that CTC induces the proliferation of PTC.

### CTC binds to miR-146 and represses its expression

**Figure 2:**
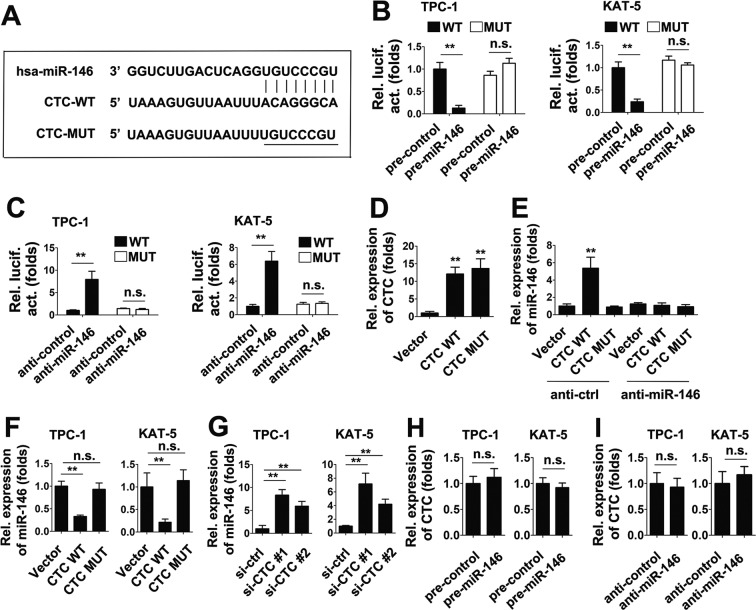
LncRNA CTC binds to miR-146 and represses its expression. (**A**) Schematic illustration of the predicted binding sites between lncRNA CTC and miR-146, and mutation of potential miR-146 binding sequence in lncRNA CTC. (**B**) TPC-1 cells (left panel) or KAT-5 cells (right panel) were co-transfected with either lncRNA CTC WT or mutants and pre-miR-146 or pre-control. Luciferase activities were measured after 48 h using a dual-luciferase assay kit and normalized to Renilla luciferase. (**C**) Experiments were performed similar to those in (**B**), except indicated anti-control or anti-miR-146 were used. (**D**) TPC-1 cells were transfected with indicated plasmid for 48 h prior to qRT-PCR assay. (**E**) TPC-1 cells were co-transfected with either lncRNA CTC WT or mutants and anti-miR-146 or anti-control for 48 h prior to pull-down assays. (**F**) TPC-1 cells (left panel) or KAT-5 cells (right panel) were co-transfected with either lncRNA CTC WT or mutants for 48 h prior to qRT-PCR assay. (**G**) Experiments were performed similar to those in (**F**), except indicated siRNAs were used. (**H**) TPC-1 cells (left panel) or KAT-5 cells (right panel) were transfected with pre-miR-146 or pre-control for 48 h prior to qRT-PCR assay. (**I**) Experiments were performed similar to those in (**B**), except indicated anti-control or anti-miR-146 were used. In the qRT-PCR experiments, the vector control was designated as 1. Bar graphs present means ± SD, n = 3 (**P < 0.01; *P < 0.05).

Because miR-146 is a potential target of CTC, we investigated whether CTC binds to miR-146. To do this, wild-type (WT) CTC were cloned into firefly luciferase reporter plasmids. One mutation was generated from WT CTC at the predicted target site to miR-146 (Fig. 2A). The luciferase activity assay showed that pre-miR-146 inhibited WT CTC luciferase activity, but not the luciferase activity of mutant CTC (Fig. 2B). Consistent with this result, anti-miR-146 induced WT CTC luciferase activity, but the mutant CTC did not (Fig. 2C). To determine whether miR-146 was indeed a target of CTC, we tested the efficacy of CTC WT and mutant expression (Fig. 2D). Pull-down assays were performed to investigate whether CTC bound miR-146. We found that WT CTC, but not mutant CTC, bound miR-146 (Fig. 2E). Anti-miR-146 abolished miR-146 precipitation (Fig. 2E). We next investigated the role of CTC on miR-146 expression. As shown Fig. 2F, CTC WT decreased miR-146 expression, whereas mutant CTC had no effect. Conversely, CTC knockdown induced miR-146 expression (Fig. 2G). Interesting, neither overexpression nor knockdown of miR-146 had an effect on CTC expression levels (Fig. 2H,I), suggesting that miR-146 was downstream of CTC. These results suggest that CTC associates with miR-146, and suppress its expression.

### CTC suppress the proliferation and invasion of PTC cells through miR-146

**Figure 3:**
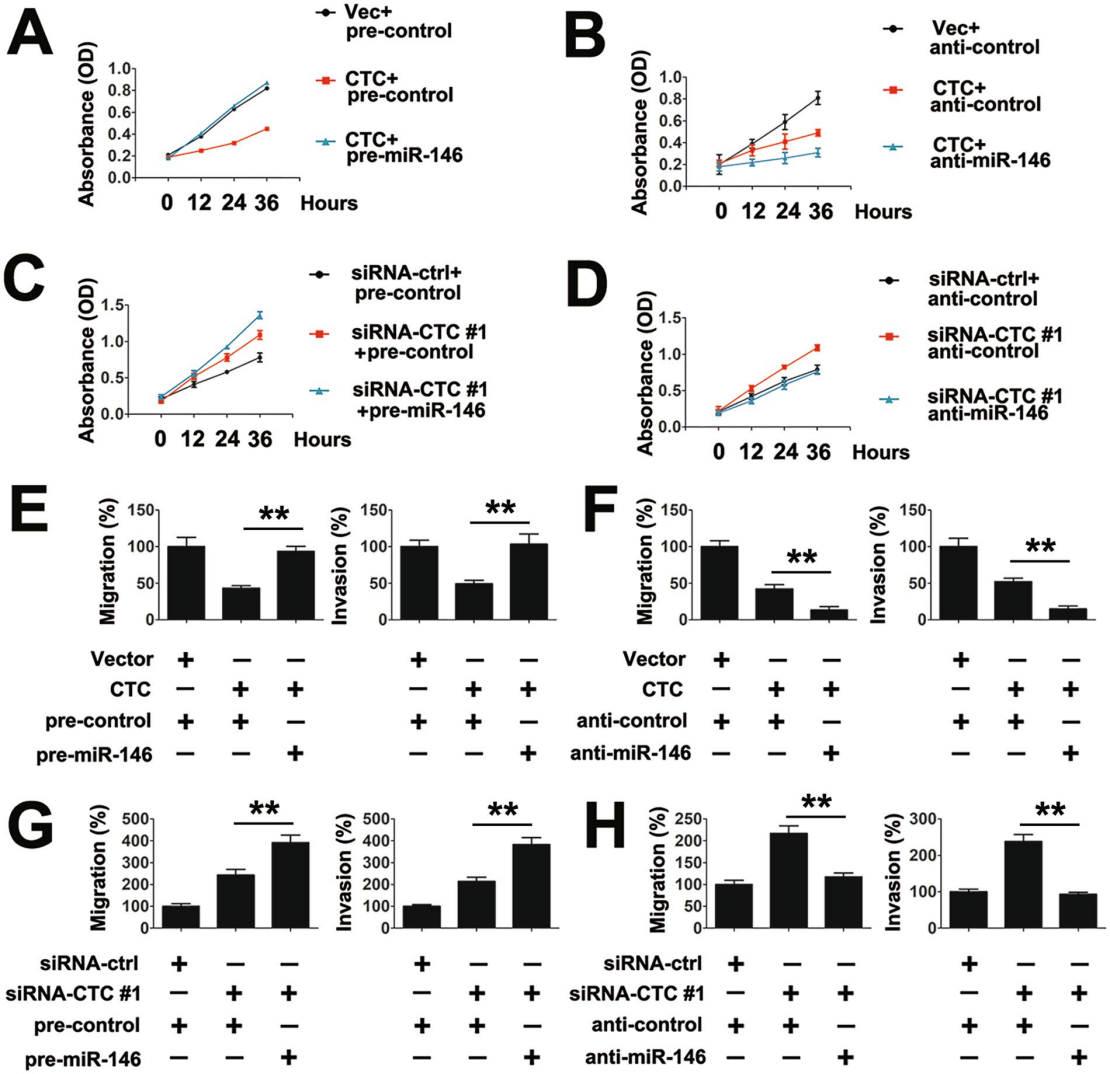
LncRNA CTC regulated PTC cells growth and motility via miR-146. (**A**) TPC-1 cells were transfected with vector control or lncRNA CTC overexpression plasmid and pre-miR-146 or pre-control for the indicated times prior to the cell proliferation assay. (**B**) Experiments were performed similar to those in (**A**), except indicated anti-control or anti-miR-146 were used. (**C**) TPC-1 cells were transfected with siRNA-ctrl or siRNA-CTC and pre-miR-146 or pre-control for the indicated times prior to the cell proliferation assay. (**D**) Experiments were performed similar to those in (**C**), except indicated anti-control or anti-miR-146 were used. (**E**) TPC-1 cells were transfected with indicated plasmid or siRNAs for 36 h prior to Transwell migration (left panel) or invasion (right panel) analyses. (**F**) Experiments were performed similar to those in (**E**), except indicated anti-control or anti-miR-146 were used. (**G**,**H**) Experiments were performed similar to those in (**E**,**F**), except indicated siRNA-control or siRNA-CTC were used. Bar graphs present means ± SD, n = 3 (**P < 0.01; *P < 0.05).

Because CTC regulates the proliferation and invasion of PTC cells and CTC binds to miR-146, we next examined the role of CTC binding of miR-146 in the proliferation of PTC cells. As shown in Fig. 3A, pre-miR-146 abolished the effect of CTC in the proliferation of PTC cells. Conversely, anti-miR-146 and the CTC overexpression plasmid synergistically inhibited PTC cell proliferation (Fig. 3B). The effect of the CTC/miR-146 signaling pathway on PTC cell proliferation was further evaluated using CTC siRNAs. Pre-miR-146 induced the effect of the CTC siRNA on the proliferation of PTC cells (Fig. 3C). Anti-miR-146 abolished the effect of CTC siRNA on the proliferation of PTC cells (Fig. 3D). Similar results were also obtained in cell migration and invasion assays (Fig. 3E–H). Together, these data demonstrate that CTC regulated the migration and invasion of PTC cells, and that miR-146 is a downstream molecule in the CTC-regulated pathway.

### CTC suppress the proliferation and invasion of PTC cells through KIT

**Figure 4:**
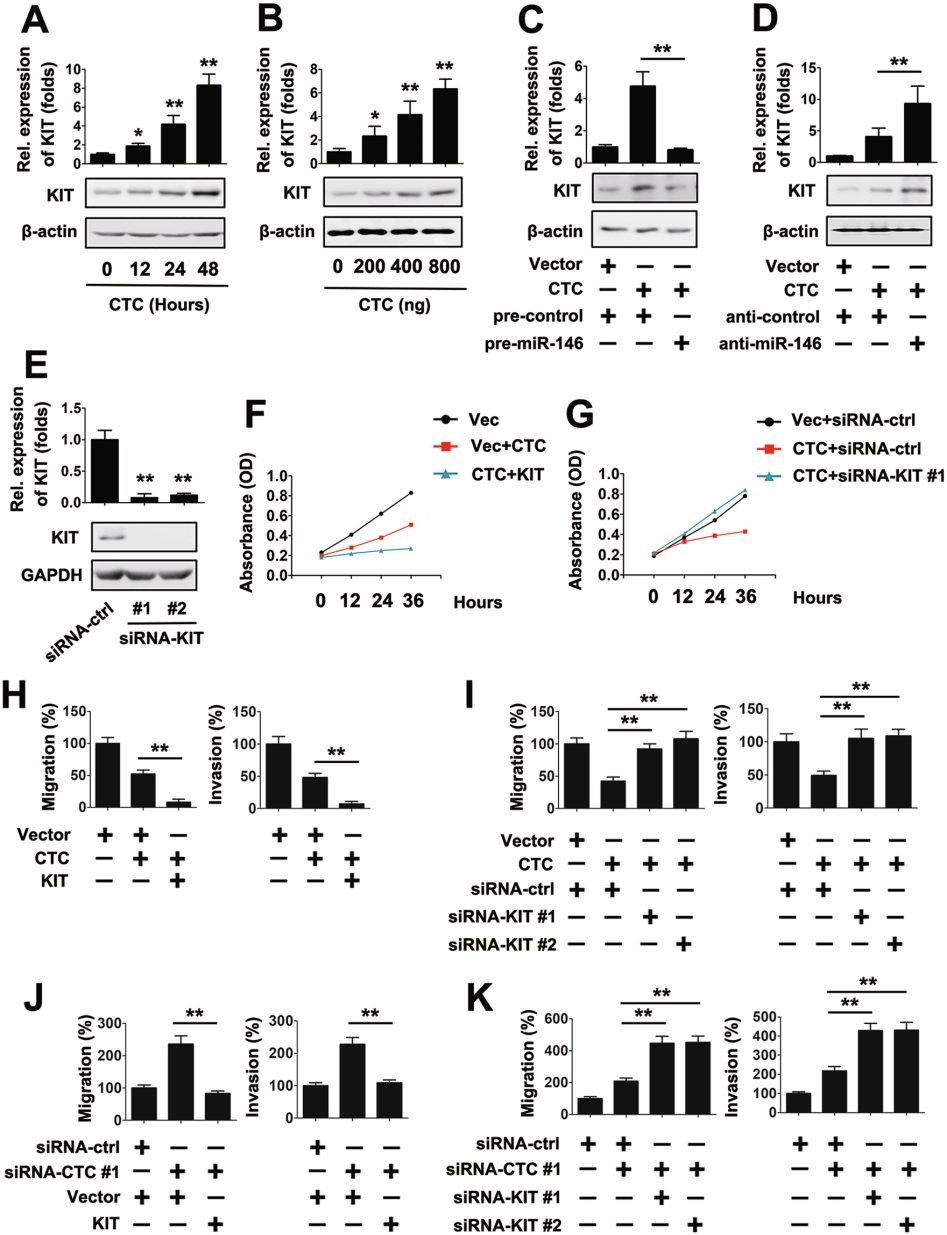
LncRNA CTC regulated PTC cells growth and motility via KIT. (**A**) TPC-1 cells (1×10^4^) were transfected with lncRNA CTC overexpression plasmid (400 ng) for the indicated times. KIT RNA levels were quantified by qRT-PCR. (**B**) TPC-1 cells (1 × 10^4^) were transfected with indicated concentration of lncRNA CTC overexpression plasmid for 24 h. KIT RNA levels were quantified by qRT-PCR. (**C**) TPC-1 cells with vector control or lncRNA CTC overexpression plasmid and pre-miR-146 or pre-control for 48 h. KIT RNA levels were quantified by real-time RT-PCR (upper panel) and protein levels of KIT were detected by western blot (lower panel). (**D**) Experiments were performed similar to those in (**C**), except indicated anti-control or anti-miR-146 were used. (**E**) TPC-1 cells with siRNA-ctrl or siRNA-KIT for 48 h. KIT RNA levels were quantified using real-time RT-PCR (upper panel) and protein levels of KIT were measured using western blot (lower panel). (**F**) TPC-1 cells were transfected with indicated plasmids for the indicated times prior to the cell proliferation assay. (**G**) Experiments were performed similar to those in (**F**), except indicated siRNA-ctrl or siRNA-KIT were used. (**H**) TPC-1 cells with vector control, KIT or lncRNA CTC overexpression plasmid for 36 h prior to Transwell migration (left panel) or invasion (right panel) assays. (**I**) Experiments were performed similar to those in (**H**), except indicated siRNA-ctrl or siRNA-KIT were used. (**J**,**K**) Experiments were performed similar to those in (**H,I**), except indicated siRNA-ctrl or siRNA-lncRNA CTC were used. All experiments were repeated at least three times with similar results. Bar graphs present means ± SD, n = 3 (**P < 0.01; *P < 0.05).

Because KIT is a target of miR-146, the influence on CTC inhibited migration and invasion of PTC cells were evaluated. First, the role of CTC on KIT expression was investigated. We found that CTC induced KIT mRNA and protein expression in a time-dependent manner (Fig. 4A). Similarly, a dose-dependent increase in KIT expression was also observed in PTC-1 cells, in which were transfected with CTC overexpression plasmid for incremental concentrations (Fig. 4A). We next determined the effect of miR-146 on CTC-regulated KIT expression. Pre-miR-146 inhibited CTC-induced KIT mRNA and protein expression levels, whereas anti-miR-146 and CTC overexpression plasmid synergistically induced KIT expression (Fig. 4C,D).

To test whether KIT affect CTC regulated PTC cell proliferation, migration and invasion, we designed two siRNA plasmids for KIT (KIT siRNA #1 and #2), and tested the efficiency of those siRNAs (Fig. 4E). MTT assays suggested that overexpression of KIT induced the effect of the CTC overexpression plasmid on the proliferation of PTC cells (Fig. 4F). By contrast, KIT siRNA #1 abolished the proliferation effect of CTC overexpression plasmid (Fig. 4G). Similarly, overexpression of KIT promoted the promigratory and proinvasive effect of CTC overexpression plasmid, whereas knockdown of KIT removed the pro-migratory and pro-invasive effects of the CTC overexpression plasmid in PTC cells (Fig. 4H,I). CTC siRNA #1 was used to determine whether CTC regulated the migration and invasion of PTC cells via KIT. Results from migration and invasion assay demonstrated that KIT overexpression abolished siRNA-CTC-induced migration and invasion of PTC cells, whereas KIT knockdown by the KIT siRNA #1 and #2 upregulated CTC siRNA #1 induced migration and invasion (Fig. 4J,K). Together, these data demonstrate that induction of KIT expression is an important event in CTC-controlled PTC cell invasion and migration.

### CTC/miR-146/KIT axis regulates PTC cell chemoresistance

**Figure 5:**
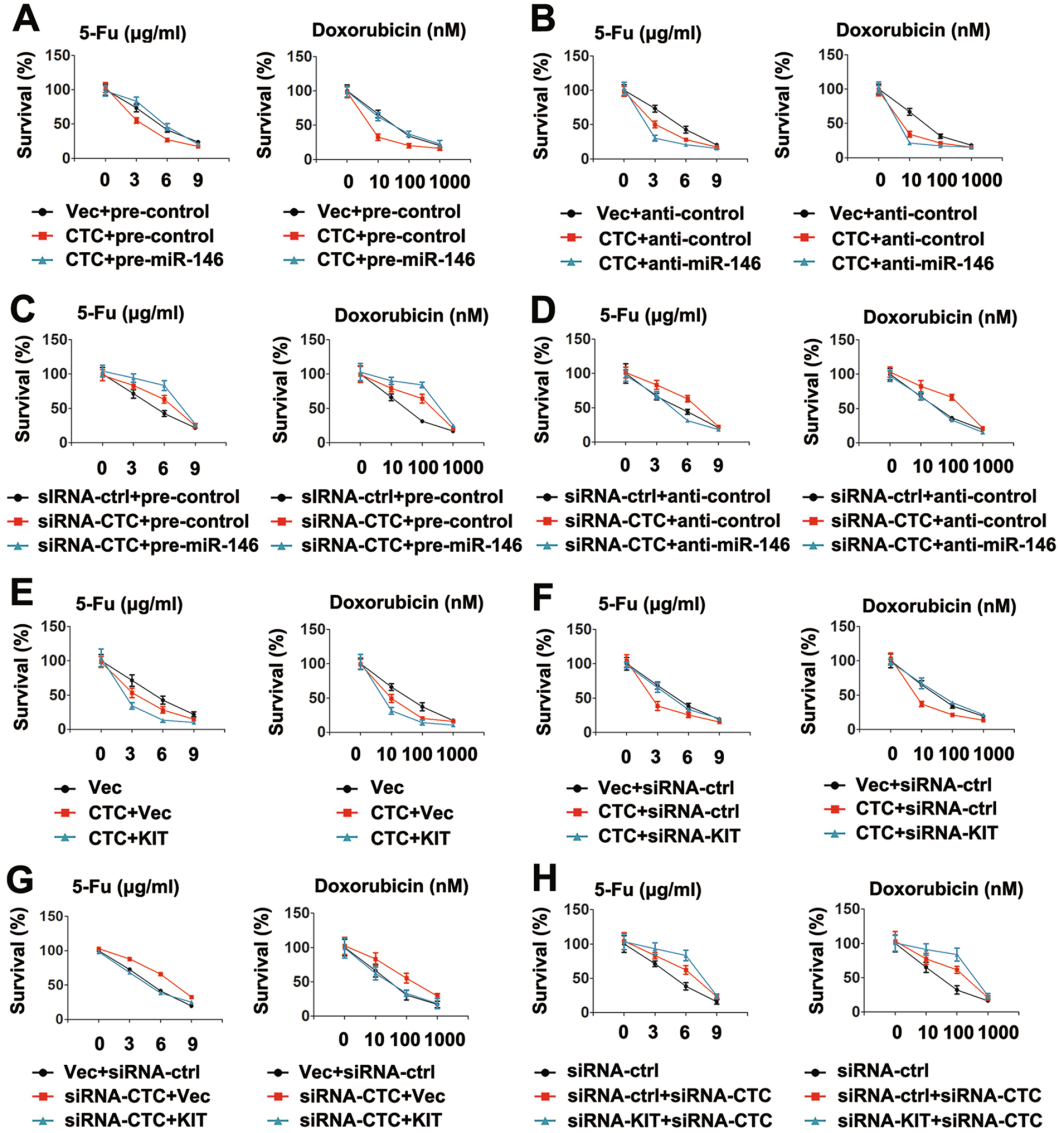
Regulation of PTC chemoresistance by lncRNA CTC/miR-146/KIT axis. (**A**) TPC-1 cells were transfected with vector control or lncRNA CTC overexpression plasmid and pre-miR-146 or pre-control for 24 h, and treated with or without indicated concentration of 5-FU (left panel) or doxorubicin (right panel) for 36 h prior to cell viability assays. (**B**) Experiments were performed similar to those in (**A**), except indicated anti-control or anti-miR-146 were used. (**C**,**D**) Experiments were performed similar to those in (**A**,**B**), except siRNA-ctrl or siRNA-lncRNA CTC were used. (**E**–**H**) Experiments were performed similar to those in (**A**–**D**), except indicated KIT overexpression plasmid or siRNA-KITs were used. Bar graphs present means ± SD, n = 3 (**P < 0.01; *P < 0.05).

Because noncoding RNAs have vital biological functions in the chemoresistance of cancer cells^29^, we next investigated whether CTC/miR-146/KIT axis plays a role in regulation of PTC cells chemoresistance. First, we investigated the role of 5-fluorouracil (5-Fu) and doxorubicin (Dox) on the expression of CTC, miR-146 and KIT. As shown in Supporting Fig. 4, 5-Fu and Dox reduced miR-146 expression and induced CTC and KIT expression. As expected, 5-Fu and Dox led to cell growth inhibition in a dose-dependent manner. Overexpression of CTC increased the sensitivity of PTC cells to 5-Fu and Dox treatment; however pre-miR-146 abrogated the effect of CTC on chemoresistance (Fig. 5A). Conversely, inhibiting miR-146 and CTC overexpression synergistically increased the sensitivity of PTC cells to 5-Fu and Dox treatment (Fig. 5B). The effect of the miR-146/CTC axis on chemoresistance was further evaluated by blocking CTC. Knockdown of CTC decreased PTC cells sensitivity to Dox and 5-Fu treatment. Overexpression of miR-146 enhanced the effect of CTC siRNA on chemoresistance, whereas knockdown of miR-146 removed the effect of CTC siRNA on chemoresistance to 5-Fu and Dox (Fig. 5C,D). Next, we evaluated whether CTC regulated cell chemoresistance via KIT. Results shown that overexpression of KIT induced CTC-inhibited chemoresistance; conversely, knockdown of KIT reduced CTC-inhibited cell chemoresistance to 5-Fu and Dox (Fig. 5E,F). Similarly, overexpression of KIT abrogated inhibition of chemoresistance by CTC (Fig. 5G). Knockdown of both CTC and KIT synergistically induced chemoresistance (Fig. 5H). Collectively, these results suggest that CTC decreases the resistance of CRC cells to chemotherapy drugs via the miR-146/KIT axis.

### Expression of CTC, miR-146, and KIT in clinical samples

**Figure 6:**
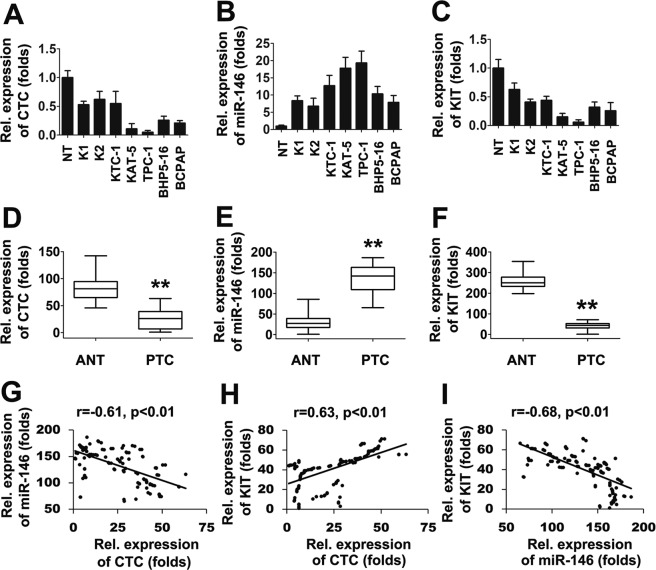
Expression lncRNA CTC, miR-146, and KIT in papillary thyroid cancer tissues. (**A**–**C**) qRT-PCR experiments analyzing the expression of lncRNA CTC (**A**), miR-146 (**B**), and KIT (**C**) in seven PTC-derived cell lines compared with five normal thyroid gland tissues (NT), whose mean value of expression was set to 1. (**D**–**F**) qPCR experiments analyzing the expression of lncRNA CTC (**D**), miR-146 (**E**), and KIT (**F**) in PTCs (n = 125) and their corresponding adjacent nontumorous tissues (ANT) (n = 125). (**G**–**I**) The relative lncRNA CTC and miR-146 levels (**G**), the relative lncRNA CTC and KIT levels (**H**) and the relative miR-146 and KIT levels (**I**) in the PTCs were subjected to Pearson’s correlation analysis. Box plots illustrate medians with 25 and 75% and error bars for 5 and 95% percentiles. For (**D**–**F**) the lowest value was designated as 1. lncRNA CTC, miR-146 and KIT data are expressed as fold induction relative to the lowest value. (**P < 0.01; *P < 0.05).

To elucidate whether CTC/miR-146/KIT axis could be an important pathway to decide the outcome of PTC, we first analyzed the expression of CTC, miR-146 and KIT in five normal thyroid (NT) tissues and seven human PTC cell lines (K1, K2, KTC-1, KAT-5, TPC-1, BHP5-16, and BCPAP). The expression levels of CTC and KIT was downregulated in PTC cell lines than NT tissues, whereas miR-146 expression was upregulated in PTC cell lines than NT tissues (Fig. 6A–C). Further studies were performed to explore clinical relevance in clinical samples by comparing CTC, miR-146 and KIT expression using qRT-PCR. As shown in Fig. 6D–F, the expression CTC and KIT was downregulated and the expression of miR-146 was upregulated in PTC tissues compared with corresponding adjacent nontumorous tissues (ANT). Moreover, the ROC curves illustrated strong separation between the PTC patients and adjacent nontumorous tissues (ANT) (Supporting Fig. 5). Correlation analysis indicated that high level CTC expression was positively correlated with high levels of KIT (Fig. 6G–I). By contrast, an inverse correlation was observed between upregulated miR-146 expression and downregulated CTC and KIT expression (Fig. 6G–I). Taken together, those results provide evidence that CTC/miR-146/KIT axis might contribute to the progression of thyroid cancer.

## Discussion

In the present study, for the first time, we described a novel signaling pathway mediating the migration and invasion of PTC cells. In this signaling pathway, CTC suppressed the migration and invasion of PTC cells. Mechanistic studies demonstrated that CTC interacts with miR-146, leading to disrupting miR-146 binding to the 3′-UTR of KIT. In addition, the CTC/miR-146/KIT axis regulated PTC chemoresistance.

Human miR-146 genes have two members, miR-146a and miR-146b, located on chromosomes 5 and 10, respectively^30^. Although miR-146a and miR-146b are encoded by different chromosomes, there are only two nucleotides that differ between the 3′ end of miR-146a and miR-146b^31^. According to limited structural differences, miR-146a and miR-146b are predicted to bind the 3′-UTR of the same genes. A previous study showed miR-146a function in inflammatory cytokine production and stem cell generation^32^. Importantly, several studies confirmed that miR-146a and miR-146b in PTC tissues could be a useful biomarker of the molecular diagnosis of PTC^25,26^. Increasing numbers of studies suggest that lncRNAs functioned as an miRNA sponge to regulate cancer development^33^. In this study, accordance with bioinformatics prediction, we found lncRNA-CTC is a potential target of miR-146. Further luciferase reporter and RNA-pull down assays confirmed that CTC sponged miR-146 to derepress PTC cell proliferation and invasion. Interestingly, CTC regulated miR-146 expression, but miR-46 did not in turn regulate CTC expression, suggesting that miR-146 is a downstream molecule in CTC-mediated signaling. In clinical samples, an inverse correlation was observed in CTC and miR-146 levels. These clinical samples further support our conclusion that there is a target relationship between CTC and miR-146. Because all clinical samples were collected at one hospital, more PTC tissues should be investigated to compensate for this limitation and strengthen the robustness of this clinical finding. MiR-146 is a key modulator of the immune response^32^; however, it remains unclear as to whether CTC participated in the immune response via miR-146. Illuminating the role of CTC in immune system could help further clarify the role of CTC function.

KIT, a member of the type III receptor tyrosine kinase family, is a tyrosine kinase receptor^34^. Downstream pathways include the MAPK and RAS and PI3-K/AKT cascades. KIT functions as an oncogene in many cancers^35^. A previous study report that miR-146 was upregulated in PTC tissues, and it regulated PTC progression by binding the 3′-UTR of KIT^25^. The activity of KIT is a hallmark in PTC; however, its biological significance remains unknown. We found that CTC regulated KIT expression via miR-146. We also found that CTC inhibited PTC cell migration and invasion via directly binding miR-146. However, we believe that this mechanism is the tip of the iceberg. One lncRNA could sponge several miRNAs simultaneously. Similarly, one miRNA can have several potential target genes. In other words, the CTC/miR-146/KIT axis may not be the only pathway targeted by CTC in PTC. Although further studies are needed to explore the potential miRNAs for CTC, we nevertheless believe that CTC might be a candidate biomarker for the progression of PTC. We also believe that interruption of CTC/miR-146-KIT signaling might point the way toward suppression PTC progression.

**Figure 7:**
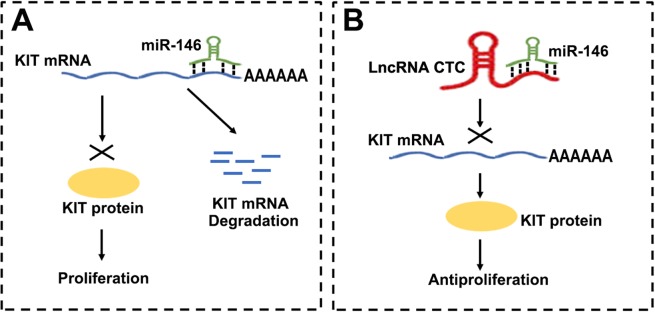
A hypothetical model for the role of lncRNA CTC in PTC cell migration and invasion.

We proposed a working model of the role of CTC in the proliferation and invasion of PTC cells (Fig. 7). In this model, miR-146 promotes PTC migration and invasion by targeting KIT 3′-UTR (Fig. 7A). However, CTC regulates proliferation and chemoresistance in PTC cells by interacting with miR-146 (Fig. 7B). As a result, miR-146 cannot target the 3′-UTR of KIT or inhibit KIT expression (Fig. 7B). Subsequently, KIT inhibits proliferation and chemoresistance in PTC cells (Fig. 7B). Our findings suggest efficient therapeutic approaches for PTC treatment.

## Methods

### Ethics statement

The study was conducted according to the principles of the 1975 Declaration of Helsinki in accordance with internationally accepted guidelines for the protection of human subjects. The collection of samples was approved by the institutional review board of Gannan Medical University and Nanjing University of Chinese Medicine, and all participants provided written informed consent to participate in the study.

### Samples and cases

PTC samples and adjacent nontumorous tissues (ANT) (located >3 cm away from the tumor) were collected from 125 patients who undergoing surgery at Gannan Medical University and Nanjing University of Chinese Medicine from April 2010 to April 2019. Two certified pathologists independently examined all PTC tissues to confirm histological subtype. ANT were confirmed by not being contaminated by cancer cells. Major inclusion criteria were as follows: (1) patients with pathologically confirmed PTC and without other malignancies; (2) patients that had not received any preoperative treatment; and (3) patients with complete clinical data, including age, sex, race, tumor size, and local invasion. Exclusion criteria were as follows: (1) patients with other kind of malignant tumors; (2) patients who were diagnosed with malignant lymphoma, poorly differentiated and anaplastic carcinomas; and (3) patients with stroke, organ transplantation, heart failure.

Tissue samples were divided into two parts. One part was used for the histologic diagnosis by two independently expert pathologists. The other part was stored in liquid nitrogen for RNA extraction. Tumors were staged according to the American Joint Committee on Cancer (AJCC) pathologic tumor-node metastasis (TNM) classification. The characteristics of patients are described in Supporting Table 1. Five normal thyroid tissues, identified as normal tissues on histological examination, were collected from the contralateral thyroid lobe of patients with benign disease.

### Cell and reagents

All human papillary thyroid cancer cell lines, including KTC-1, K1, KAT-5, K2, TPC-1, BCPAP, and BHP5-16, were purchased from Cell Bank of the Chinese Academy of Science (Wuhan, China). All cell lines were cultured in Dulbecco’s modified Eagle’s medium (DMEM) (Gibco BRL, Grand Island, NY) containing with fetal bovine serum (Gibco BRL, Grand Island, NY) (10%), penicillin (100 U/mL) and streptomycin sulfate (100 µg/mL). Cells were maintained at 37 °C in a humidified atmosphere of 5% CO_2_ and 95% air.

TRIzol, Lipofectamine-3000, and Enzyme MIX were purchased from Invitrogen (Basel, Switzerland). Monoclonal antibodies (Abs) against human KIT (ab32363, 1:1000), β-actin (ab8226, 1:5000) and GAPDH (ab181602,1:5000) were purchased from Abcam. SiRNAs and negative control (siRNA-control) were purchased from Qiagen (Germany). Efficiency of RNA interferences was determined using qRT-PCR or western blotting analyses (Fig. 4E and Supplementary Fig. 3). MiR-146 precursor (Pre-miR-146), miR-146 oligonucleotide (anti-miR-146), and its corresponding control (pre-control and anti-control) were purchased from Ambion (Austin, TX, USA). Unless specified otherwise, all biochemical reagents were purchased from Sigma-Aldrich (St. Louis, MI).

### Tumor formation in nude mice

For each male BALB/c nude mouse (Ten 6-week-old), approximately 2×10^6^ shRNA-Ctrl cells and siRNA-CTC cells were injected into the scapulae. The tumor volume was measured every 3 days by calculating tumor width and length. The formula was as follows: tumor volume = 1/2 length × width^2^. Animals were sacrificed by anesthesia 5 weeks after treatments, and the tumors were collected for further analysis. All animal manipulated according to the National Institutes of Health Guide for the Care and Use of Laboratory Animals. Animal manipulated according to the protocols approved by the institutional animal care and use committee of Gannan Medical University.

### Cell proliferation assays

Cell proliferation activity was determined by using the Cell Counting Kit-8 (CCK-8) in accordance with the manufacturer’s instructions (Dojindo Chemical Laboratory, Kumamoto, Japan). The absorbance (450 nm) were quantified by Power Wave XS microplate reader (Omega Bio-Tek, Inc., Norcross, GA, USA). Each group has five replicates. All experiments were independently repeated three times.

### Migration and invasion assays

Migration and invasion assays were performed as described^36^. Briefly, cells were collected and plated the upper chamber (Corning) coated with (invasion) or without (migration) Matrigel-coated membrane matrices (BD Biosciences). The lower chamber was added medium with 10% FBS. Twenty-four hours later, cells were fixed with 4% formaldehyde and stained with crystal violet (Merck, Darmstadt, Germany). Cells were counted under a microscope (Olympus, Tokyo, Japan) at 200x magnification. The number of cells was the average value from six representative fields.

### Transfection and luciferase reporter gene assays

Cells were grown to 80% confluence prior to transfection using Lipofectamine 3000 in accordance with the manufacturer’s instructions (Invitrogen). Twenty-four hours later, cells were serum-starved for other 24 hours. Luciferase activity was measured using a dual-specific luciferase assay kit (Promega) (Madison, WI), and Renilla luciferase activities was used as internal control.

### Quantitative real-time RT-PCR (qRT-PCR)

Mature microRNAs (miRNAs) and mRNA levels was detected by using qRT-PCR assays as described^37^. For miRNAs, total RNA was isolated using a mirVana miRNA isolation kit (Ambion). Two micrograms of total RNA were reverse transcribed using Bulge-Loop mature miRNA-specific reverse transcription primers (Ambion). To test the expression levels of miRNAs, miRNA analysis kits (Ambion) was used according to the manufacturer’s instructions. U6 snRNA was used as the internal control.

For cellular mRNAs, total RNA was extracted using TRIzol reagent according to the manufacturer’s instructions (Invitrogen, Basel, Switzerland). One microgram of total RNA was used as a template for reverse transcription by random primers and M-MLV Reverse Transcriptase (Promega). We performed qRT-PCR using the SYBR Green system (Applied Biosystems), with GAPDH as the internal control. The procedure was performed on a LightCycler 480 (Roche), and sequencing was used for the verification of qRT-PCR products. Primers used this study are listed in Supporting Table 2.

### Western blot analysis

Western blot analyses were performed as described^36^. Briefly, cells were harvested and lysed in radio immunoprecipitation assay (RIPA) buffer (Cell Signaling Technology, 9800). Protein concentrations were quantified using BCA assays (Cell Signaling Technology, 7780). Cell lysates (40 μg) were electrophoresed using 12% SDS-PAGE, and were then transferred to a nitrocellulose membranes (Bio-Rad) that were subsequently blocked with 5% (w/v) nonfat dried milk. One hour later, blots were incubated with primary antibodies, as indicated in the figures, overnight at 4 °C. Subsequently, immunocomplexes were incubated with horseradish peroxidase-linked secondary antibodies (Jackson ImmunoResearch) and then blots were developed using an enhanced chemiluminescence system (GE Healthcare).

### Statistical analysis

GraphPad Prism 5 software (GraphPad Software, La Jolla, CA, USA) was used for statistical analyses. Parametric data was analyzed by using a two-tailed t-test. Nonparametric data was analyzed by using the Mann–Whitney U test. Means were illustrated using a histogram with error bars representing presented as mean ± SD or mean ± SEM, and P < 0.05 was considered statistically significant.

## Supplementary information

Supplemental information.

## Data Availability

All data generated or analyzed during this study are included in this article.
