# Supplementary material for: Long Noncoding RNA CTC Inhibits Proliferation and Invasion by Targeting miR-146 to Regulate KIT in Papillary Thyroid Carcinoma
Source: Sci Rep. 2020 Mar 12;10:4616. doi: 10.1038/s41598-020-61577-z (PMC7067803; doi:10.1038/s41598-020-61577-z)
Supplement: Supplementary file 1 — Supplemental information. [file 41598_2020_61577_MOESM1_ESM.docx]

**Supplemental Information**

**Long Noncoding RNA CTC Inhibits Proliferation and Invasion by Targeting miR-146 to Regulate KIT in Papillary Thyroid Carcinoma**

Baochun Liao^1^, Shi Liu ^2^, Jiafeng Liu^1^, Pulusu Ajay Kumar Reddy^1^, Yong Ying^1^, Yang Xie^1^, Jianhua Wang^3,*^, Xiangtai Zeng^1,*^


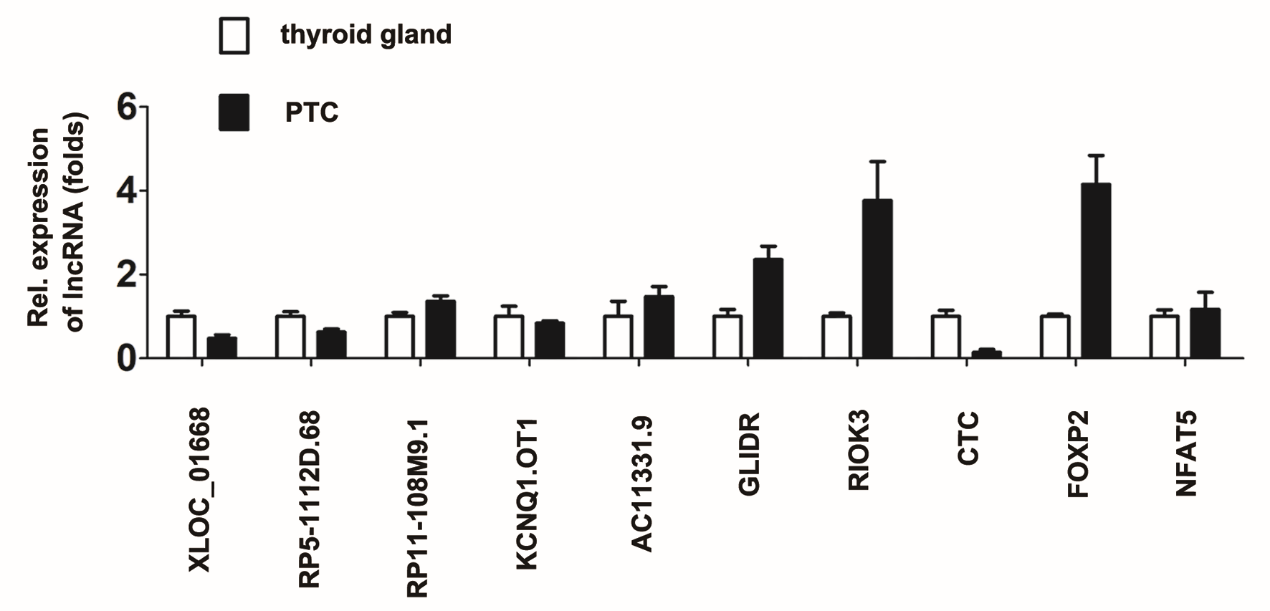


**Supporting Fig. 1.** **Screening for the expression of potential lncRNAs that can interact with miR-146 in thyroid and PTC.** qRT-PCR experiments analyzing the expression of indicated lncRNAs in PTC in comparison with normal thyroid samples. Bar graphs represent the means ± SD, n = 3 (**P < 0.01; *P < 0.05).


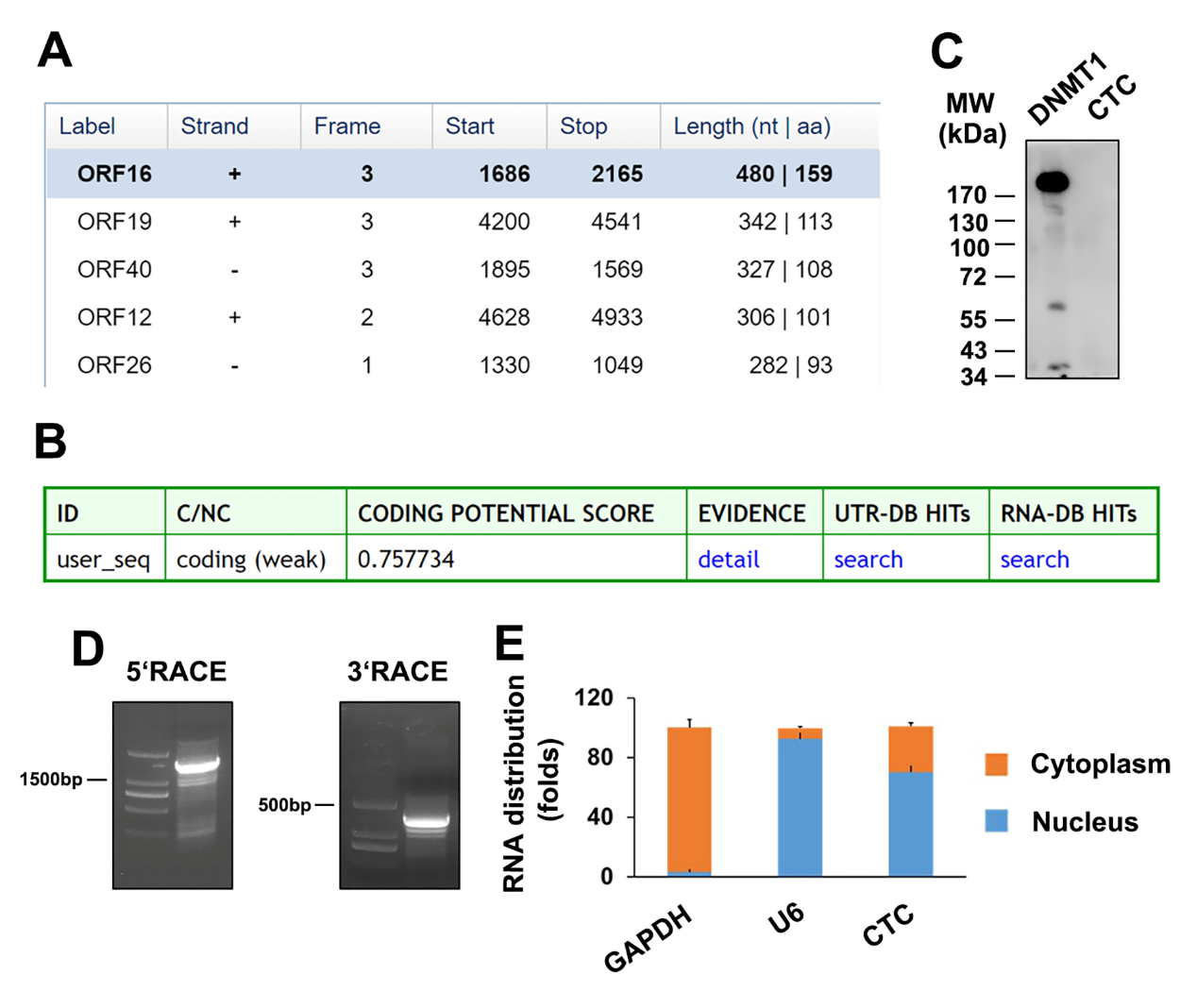


**Supporting Fig. 2. Analyze the coding potential of lncRNA-CTC.** (A) The potential ORF analysis for each transcript of lncRNA-CTC by Open Reading Frame Finder (<https://www.ncbi.nlm.nih.gov/orffinder/>). (B) The CPC scores for lncRNA-CTC. (C) LncRNA-CTC were cloned into Pnl vector with N-terminal Flag tag. The plasmids were transfected into 293 T cells for 48 h. Cell lysates were harvested and subjected to Western blotting with anti-Flag. Flag-DNMT1 served as a positive control. (D) Agarose gel analysis of 5’ and 3’ RACE PCR products of lncRNA-IUR-5. (E) qRT-PCR was performed to examine cytoplasmic or nuclear lncRNA-CTC expression in PTC cells. GAPDH served as a cytoplasmic control, and U6 as a nuclear control. All experiments were repeated at least three times with consistent results. Bar graphs represent the means ± SD, n = 3 (**P < 0.01; *P < 0.05).


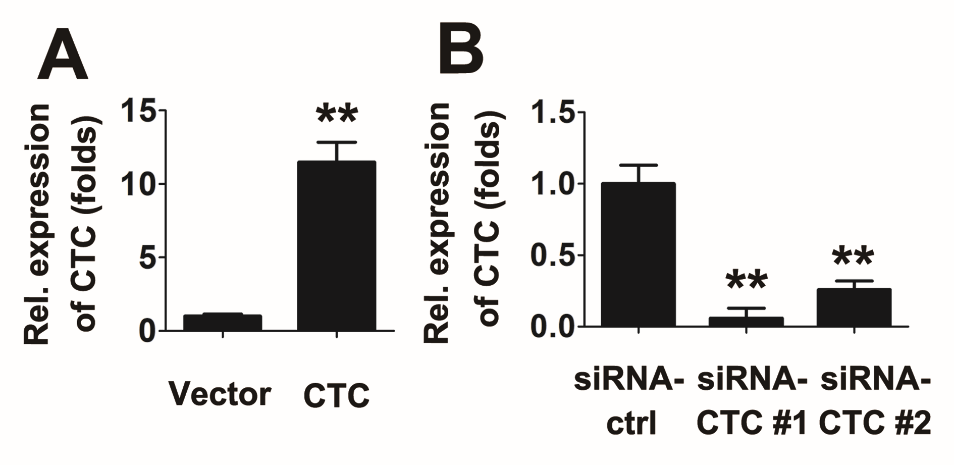


**Supporting Fig. 3. Determination of the efficiency of CTC** **overexpression plasmid and siRNAs.** (A) TPC-1 cells were transfected with vector control or lncRNA CTC overexpression plasmid for 48 h prior to qRT-PCR assay. (B) Experiments were performed similar to those in (A), except indicated siRNAs were used. Bar graphs represent the means ± SD, n = 3 (**P < 0.01; *P < 0.05).


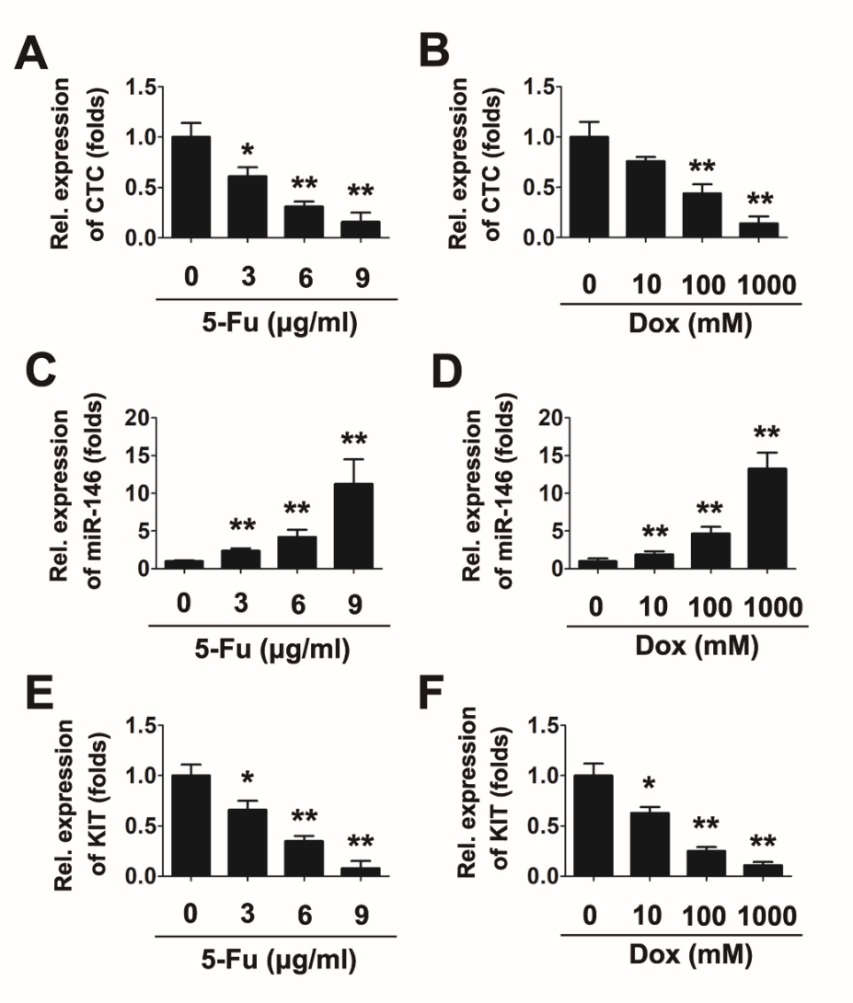


**Supporting Fig. 4.** **Determination of the role of 5-FU and Dox on the expression of CTC,** **miR-146 and KIT.** (A and B) TPC-1 cells treated with or without indicated concentration of 5‑FU (A) or doxorubicin (B) for 36 h prior to qRT-PCR assays. (C-F) Experiments were performed similar to those in (A and B), except miR-146 (C and D) and KIT (E and F) expression was determined by qRT-PCR assays. Bar graphs represent the means ± SD, n = 3 (**P < 0.01; *P < 0.05).


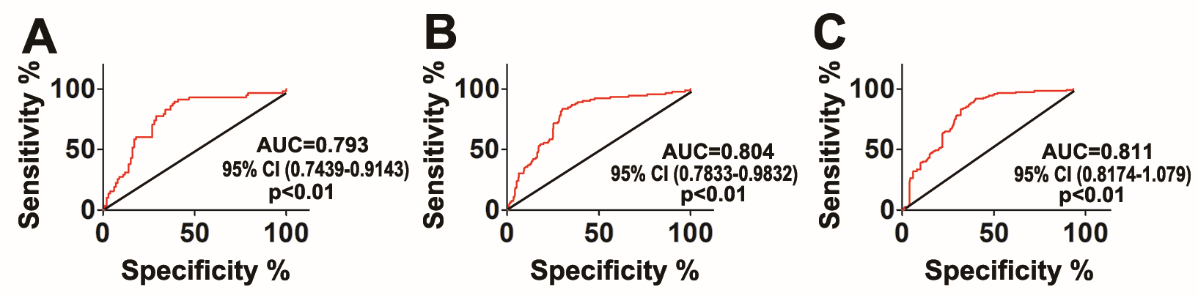


**Supporting Fig. 5. The ROC analysis of lncRNA CTC (A), miR-146 (B), and KIT (C) for PTC patients in 125-pair tissues.**

**Supplemental Table 1: Correlation of CTC, miR-146 and KIT expression with** **clinicopathologic features in papillary thyroid cancers (PTC).**

| **Clinicopathologic parameters** | **Case no.** | **CTC expression (folds)** | **P value** | **miR-146 expression(folds)** | **P value** | **KIT expression (folds)** | **P value** |
| --- | --- | --- | --- | --- | --- | --- | --- |
| **Age** |  |  |  |  |  |  |  |
| ≤50 | 62 | 22.14±5.17 | ns | 130.34±13.02 | ns | 40.51±4.57 | ns |
| ＞50 | 63 | 26.17±4.99 |  | 137.25±13.32 |  | 41.49±3.22 |  |
| **Tissue type** |  |  |  |  |  |  |  |
| Normal tissue | 125 | 81.48±10.41 | <0.01 | 28.62±3.64 | <0.01 | 255.04±26.15 | <0.01 |
| Carcinoma | 125 | 24.43±6.37 |  | 133.81±14.17 |  | 41.15±5.17 |  |
| **Sex** |  |  |  |  |  |  |  |
| Male | 65 | 23.74±4.09 | ns | 135.24±12.34 | ns | 42.15±4.74 | ns |
| Female | 60 | 25.48±3.34 |  | 13219±13.07 |  | 39.98±4.55 |  |
| **Tumor size** |  |  |  |  |  |  |  |
| ≤5cm | 59 | 38.18±4.36 | <0.01 | 84.35±7.55 | <0.01 | 57.88±6.39 | <0.01 |
| ＞5cm | 66 | 10.24±2.74 |  | 183.49±15.91 |  | 24.15±2.15 |  |
| **TNM stage** |  |  |  |  |  |  |  |
| Ⅰ | 29 | 45.93±4.02 | <0.05 | 34.24±3.15 | <0.05 | 8.26±1.02 | <0.05 |
| Ⅱ | 31 | 28.15±2.33 |  | 63.48±5.14 |  | 18.29±1.97 |  |
| Ⅲ | 32 | 16.24±2.04 |  | 134.15±11.74 |  | 41.25±3.62 |  |
| Ⅳ | 33 | 7.26±1.14 |  | 304.97±28.36 |  | 97.18±8.29 |  |
| **Lymph nodemetastasis** |  |  |  |  |  |  |  |
| Negative | 61 | 40.25 ±5.36 | <0.01 | 77.58±6.34 | <0.01 | 62.28±6.33 | <0.01 |
| Positive | 64 | 8.15±2.36 |  | 190.11±17.41 |  | 20.74±2.15 |  |
| **Distant metastasis** |  |  |  |  |  |  |  |
| Negative | 63 | 41.42±4.36 | <0.01 | 71.96±5.06 | <0.01 | 63.91±5.71 | <0.01 |
| Positive | 62 | 7.93±1.06 |  | 196.4±19.35 |  | 18.29±2.09 |  |

**Supplemental Table 2: Primers Used in qRT-PCR**

| **Target gene** | **5’primer (5’ to 3’)** | **3’primer (5’ to 3’)** |
| --- | --- | --- |
| **GAPDH** | GGAAGGTGAAGGTCGGAGTCAACGG | CTCGCTCCTGGAAGATGGTGATGGG |
| **U6** | CCCCAGTCACCAGTTGCTCG | AGGGCTGCTGGAAGTTGGAC |
| **CTC** | GGACCTTTGCTTGGGATGCG | CCTGTGACTAACGGCTGGAA |
| **KIT** | TTTCCTTGTTGACCGCTCCT | AGCACTGACTTGCCCTCCTG |
| **MiR-146** | CctggcacTGAGAACTGAAT | ggcaccagaactgagtccac |
